# Cannabidiol regulates apoptosis and autophagy in inflammation and cancer: A review

**DOI:** 10.3389/fphar.2023.1094020

**Published:** 2023-01-23

**Authors:** Ze Fu, Peng-Yue Zhao, Xing-Peng Yang, Hao Li, Shi-Dong Hu, Ying-Xin Xu, Xiao-Hui Du

**Affiliations:** ^1^ Medical School of Chinese PLA, Beijing, China; ^2^ Department of General Surgery, First Medical Center of Chinese PLA General Hospital, Beijing, China

**Keywords:** cannabidiol, ECS, apoptosis, autophagy, inflammation, cancer

## Abstract

Cannabidiol (CBD) is a terpenoid naturally found in plants. The purified compound is used in the treatment of mental disorders because of its antidepressive, anxiolytic, and antiepileptic effects. CBD can affect the regulation of several pathophysiologic processes, including autophagy, cytokine secretion, apoptosis, and innate and adaptive immune responses. However, several authors have reported contradictory findings concerning the magnitude and direction of CBD-mediated effects. For example, CBD treatment can increase, decrease, or have no significant effect on autophagy and apoptosis. These variable results can be attributed to the differences in the biological models, cell types, and CBD concentration used in these studies. This review focuses on the mechanism of regulation of autophagy and apoptosis in inflammatory response and cancer by CBD. Further, we broadly elaborated on the prospects of using CBD as an anti-inflammatory agent and in cancer therapy in the future.

## Introduction

Cell death plays an important role in physiologic and pathophysiologic processes, implying that the pathogenesis of human diseases is closely related to this process. Cell death can be broadly divided into two categories, namely programmed cell death (PCD) and simple necrosis. PCD refers to the ontogenetic, preprogrammed, and tightly controlled death of certain cells in a multicellular organism. PCD includes apoptosis, programmed necrosis, and pyroptosis. Apoptosis is usually caused by some proapoptotic stimuli, such as endoplasmic reticulum stress and accumulation of reactive oxygen species. These stimuli activate several caspases and eventually cause a series of irreversible changes in cells. Morphologically, apoptosis is characterized by cell shrinkage, chromatin condensation, blebbing, and the formation of apoptotic bodies (Kishino et al., 2019; Hu et al., 2020). Despite this, the cell membrane remains intact and no inflammation occurs (Zhang et al., 2018). The process of apoptosis avoids the occurrence of inflammatory reactions and tissue damage to achieve a steady state while maintaining an internal environment and protecting the host.

Autophagy is a phylogenetic degradation process that depends on the formation of specialized membrane structures including phagosomes, autophagosomes, and autolysosomes. Autophagy plays a complex role in maintaining health and developing diseases (Levine and Kroemer, 2019). Eukaryotic cells maintain homeostasis and renew themselves through autophagy, which is an evolutionarily conserved mechanism (Hao et al., 2022). Autophagy can be divided into macroautophagy, microautophagy, and chaperone-mediated autophagy according to the different routes of cellular material transported to lysosomes; macroautophagy is the most common type among them. Autophagy is thought to be a major factor in determining the fate of the cells and can trigger apoptosis. This implies that autophagy can also represent a form of programmed cell death known as autophagic cell death or type II programmed cell death. Three sequential steps are involved in intact autophagy: induction, autophagosome formation, and autophagosome-lysosome fusion and degradation. Bulk and selective are the two modes of autophagy (Gatica et al., 2018), contributing to metabolic adaptation and cellular homeostasis, respectively (Kaur and Debnath, 2015).

**FIGURE 1:**
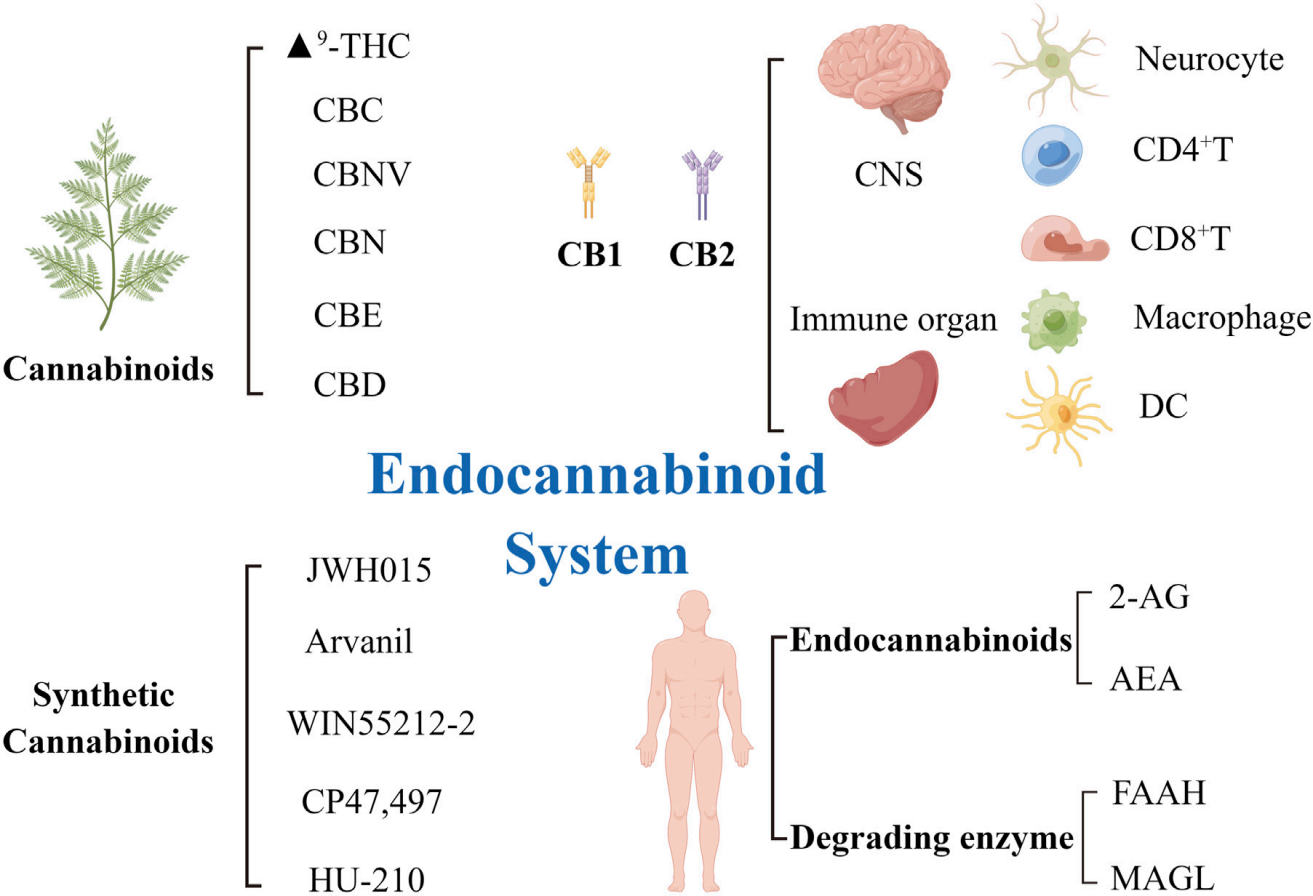
The elements of the endocannabinoid system and receptors distribution. ECS is composed of its two main endocannabinoids AEA and 2-AG, which activate their primary targets CB1 and CB2. AEA was degraded by FAAH enzyme and 2-AG by MAGL enzyme. Six plant cannabinoids (phytocannabinoids), Δ9-THC, CBC, CBNV, CBN, CBE, and CBD, and of five synthetic cannabinoids, JWH015, Arvanil, WIN55212-2, CP47/497, HU-210. CB1 receptors are mainly distributed in the central nervous system and the surface of neurocyte. CB2 receptors are mainly distributed in the peripheral immune system and on the surface of immune cells, such as CD4+T cells, CD8+T cells, macrophages and dendritic cells. AEA, arachidonoylethanolamine; 2-AG, 2-arachidonoylglycerol; Δ9-THC, Δ9-tetrahydrocannabinol; CB1/2, cannabinoid receptor1/2; CBC, cannabichromene; CBD, cannabidiol; CBE, cannabielsoin; CBN, cannabinol; CBNV, cannabinolivarin; ECS, endocannabinoid system; FAAH, fatty acid amide hydrolase; MAGL, monoacylglycerol lipase.

Autophagy is closely associated with several human diseases, such as cancer, neurodegenerative diseases, and infectious diseases (Chung et al., 2017; Padman et al., 2019; Amorim et al., 2022). The endocannabinoid system is a naturally occurring lipid signaling system intricately related to a range of physiologic and disease processes. The endocannabinoid system comprises endocannabinoid receptors, endocannabinoids, and metabolic enzymes responsible for regulating the synthesis and decomposition of ligands. The endocannabinoids mainly exist in two forms in the body, arachidonoylethanolamine (AEA) and 2-arachidonoylglycerol (2-AG). Endocannabinoid receptors mainly exist in mammalian tissues with two types of G protein-coupled receptors, CB1 and CB2. CB1 is mostly distributed in the central nervous system (Ghosh et al., 2021; Pant et al., 2021), including the amygdala, cortex, hypothalamus, hippocampus, and cerebellum; the proportion of CB1 in these locations is higher than in other locations. CB2 is mainly expressed in peripheral immune cells (lymphocytes, macrophages, and splenocytes). The endocannabinoid system is important in the regulation of the autonomic nervous system, immune system, and peripheral microcirculation. The theory of clinical endocannabinoid deficiency syndrome (CEDS), proposed by Russo, suggests that some chronic diseases may occur because of a lack of endocannabinoid signaling (Silver, 2019). Therefore, whether the supplementation of exogenous cannabinoids can maintain the homeostasis of the body and improve the prognosis of some diseases is worth examining. Tetrahydrocannabinol (THC) and CBD are the most common cannabinoids used in medical cannabis preparations. However, the use of THC, which has psychoactive features, has adverse effects on the nervous system. THC-treated mice showed obvious but transient neurological changes, slow movement and breathing, hyperactivity, secondary immune dysfunction, and impaired ability to eliminate infection (Fitton and Pertwee, 1982)]. In contrast, CBD is a non-psychoactive terpenoid and has a wide range of biological effects, including anti-inflammatory, anticancer, and neuroprotective effects. Therefore, CBD has been the main focus of research on bioactive compounds for treating psychological disorders. This review aims to describe the critical role of CBD in the regulation of apoptosis and autophagy. This information may be useful in analyzing the prospects of using CBD as an anti-inflammatory agent and in innovative cancer therapy for clinical use (Figure 1).

### Effect of apoptosis on the pathophysiology of inflammatory diseases

**FIGURE 2:**
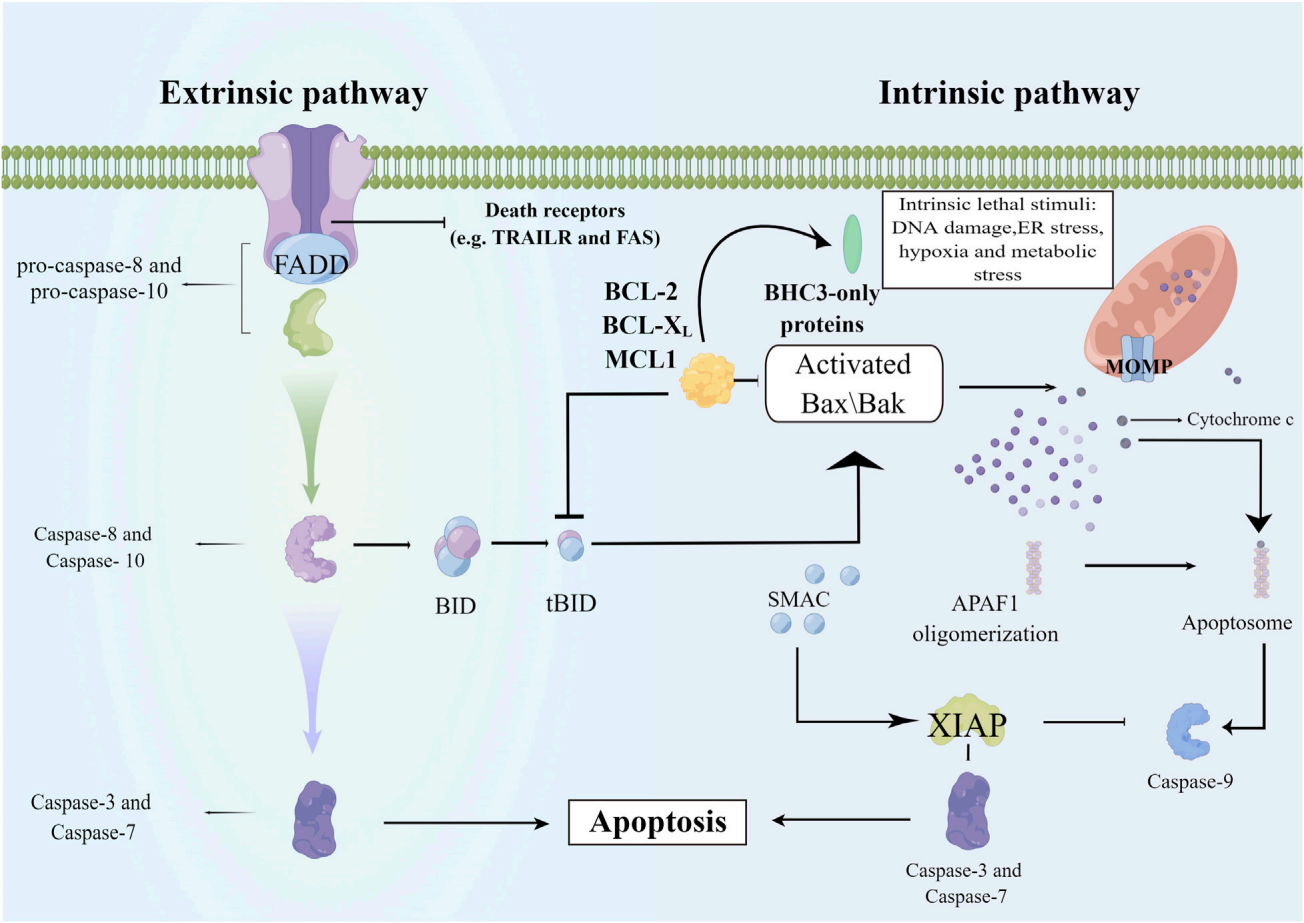
The mechanism of cell apoptosis. The extrinsic pathway involves the recruitment and activation of procaspase-8, and activated caspase-8 then directly activates the effector caspases such as caspase-3 to initiate the execution process. The intrinsic apoptotic pathway is mediated by the cleavage of BID (BH3 interacting domain death agonist), a BCL-2 homology 3 (BH3)-only protein. Truncated BID (tBID) subsequently translocates to the mitochondria and activates the BCL-2 family members BAX and BAK. Upon activation, BAX and BAK induce mitochondrial outer membrane permeabilization and the release of proapoptotic mitochondrial contents into the cytoplasm, such as cytochrome c. Released cytochrome c binds APAF1, and APAF-1 recruits procaspase-9 through the CARD-CARD interaction and forms the apoptosome, leading to proximity-induced activation of caspase-9, which in turn cleaves and activates effector caspases (Members of the IAP family including XIAP negatively regulate caspase activation and they can be inactivated by SMAC). APAF1, apoptotic protease activating factor-1; Bax/Bak, a proapoptotic member of Bcl-2 family; BID, BID protein; FADD, Fas associated death domain; MCLl, myeloid cell leukemia-1; MOMP, mitochondrial outer membrane permeabilization; SMAC, second mitochondria-derived activator of caspases; X1AP, X-linked inhibitor of apoptosis protein.

Apoptosis is regarded as a non-inflammatory process, but the lytic nature of necrosis triggers the release of intracellular damage-associated molecular patterns and eventually leads to inflammation. Apoptotic signaling pathways are of two types: mitochondrial-mediated (intrinsic) and death receptor-mediated (extra) pathways. In the death-receptor-mediated pathway, FasL binds to Fas, leading to the recruitment of various proteins and activation of downstream caspase-8, caspase-7, and caspase-3. The intrinsic pathway of apoptosis is regulated by the B-cell lymphoma-2 (Bcl-1) family of antiapoptotic (such as Bcl-2 and Bcl-xL) and proapoptotic proteins (such as Bad, Bid, Bax, and Bim). The caspase-8-mediated cleavage of Bid into its active form (tBid) leads to mitochondrial dysfunction and cytochrome c release, which activates downstream caspase-9 and caspase-3, thereby mediating cell death. Crosstalk between extrinsic and intrinsic pathways can occur because of the similar mechanism of eliciting apoptosis (Zhang et al., 2018). These pathways are initiated by effector caspases and result in DNA fragmentation, cytoskeletal reorganization, cytoplasmic condensation, and the formation of apoptotic bodies (Figure 2).

Several types of cells are affected in the course of inflammation. One of the important mechanisms of inflammatory response involves the imbalance of apoptosis regulation that leads to abnormal distribution or quantity of cell types. Consequently, it is vital to clarify the relationship between apoptosis and inflammation, especially in the context of the various cell types involved. Apoptosis or programmed cell death is essential for cell homeostasis, injury repair, immune tolerance, and inflammation resolution in multicellular organisms (Rodionov et al., 2022). When apoptosis occurs, the apoptotic cells are cleared by specialized phagocytes (macrophages and immature dendritic cells) or other cells (such as endothelial and mesenchymal cells). Apoptosis inhibits the transcription of pro-inflammatory cytokine genes, downregulates the expression of CD40L and CD40, silences CD4^+^ and CD8^+^ T cells, and promotes the secretion of anti-inflammatory cytokines (TGF-β, IL-10, and IL-13). In the acute phase of sepsis or acute organ injury, excessive inflammation induces and aggravates apoptosis (Chen et al., 2016a). Further, apoptosis continues to aggravate the inflammatory injury effect, eventually causing irreversible organ function damage. A report on acute liver injury suggested that excessive generation of reactive oxygen species not only directly damages cells but also recruits inflammatory cells and activates pro-inflammatory cytokines, both of which induce mitochondrial apoptosis (Chen et al., 2016b). In a study on microvascular endothelial cell injury, oxidative stress markedly activated the cAMP response element binding protein, which enhanced the transcription and expression of IP3R and VDAC, resulting in increased Ca2+ content and the release of cytochrome c into the cytoplasm, thereby activating the mitochondria-dependent death pathway (Zhu et al., 2018). Excessive oxidative stress, increase in inflammatory responses, and cellular calcium overload are upstream activators of mitochondria-dependent apoptosis.

Improper clearance of apoptotic cells results in the occurrence and progression of several human chronic inflammatory diseases such as autoimmune diseases, including rheumatoid arthritis (RA) and systemic lupus erythematosus (SLE), neurological disorders, obesity, type 2 diabetes, and atherosclerosis. Correspondingly, disruption of inflammatory reaction also disturbs cell apoptosis. While treating autoimmune disease, the use of certain drugs can promote caspase-dependent cell apoptosis and reduce IL-10 and IL-12 secretion. The use of non-steroidal anti-inflammatory drugs for treating inflammatory diseases may be associated with the activation of peroxigenic proliferator-activated receptor (PPAR)γ, which ultimately induces apoptosis. In patients with SLE, the accumulation of myeloid-derived suppressor cells (MDSCs) is positively correlated to disease activity. Hydroxychloroquine may relieve the symptoms of SLE inflammation by regulating the expression of CD81 in MDSCs and inducing their apoptosis (Ni et al., 2022). Taken together, promoting the apoptosis of key cells in inflammatory diseases and reducing the secretion of inflammatory cytokines are crucial in alleviating inflammatory responses. The efficient execution of apoptotic cell death followed by the clearance of the debris by phagocytes is a key mechanism in maintaining tissue homeostasis.

### Autophagy is connected to the process of inflammatory disease

Autophagy is a metabolic, cytoplasmic quality control, and homeostasis-maintaining process having cytoprotective, tissue-protective, and anti-inflammatory effects. Autophagy contributes to the removal of cell-damaging irritants or invading pathogens in adaptive and innate immune cellular processes, asepsis, and infection-related inflammation. The close association between inflammatory diseases (dysregulation of inflammatory responses in multiple diseases leading to histopathologic changes in several human organs) and alterations in autophagy was first identified from the studies on the association between genetic polymorphisms and disease susceptibility in humans [ (Wellcome Trust Case Control Consortium, 2007). Genetic susceptibility to various diseases, such as asthma, RA, SLE, multiple sclerosis, and other neurological disorders, has been linked to mutations in autophagy genes (Orozco et al., 2011; Ramos et al., 2011; Martin et al., 2012; Schuster et al., 2015). Inflammasomes are cytoplasmic high-molecular-weight protein complexes that activate caspase-1 in response to inflammation triggered by microbial infection or injury. They comprise nucleotide-binding oligomerization domain-like receptor (NLR) family protein, adaptor apoptosis-related spot-like protein containing caspase-recruitment domain, and effector protease caspase-1 (Cheng et al., 2022). Activated caspase-1 converts pro-inflammatory cytokines IL-1β and IL-18 precursors into their biologically active forms and cleaves GSDMD to induce pyroptosis. Autophagy can eliminate agonists (such as damaged mitochondria) and components of NLRP3 inflammasomes and regulate inflammation. Autophagy removes damaged mitochondria and a complete autophagy process is necessary to prevent the release of endogenous inflammasome agonists (reactive oxygen species and oxidized mitochondrial DNA) (Zhou et al., 2011). Currently, several authors have linked the promotion of autophagy with the prevention of inflammasome activation. MEFV/TRIM20, a member of the TRIM protein family, recognizes the pro-CASP1, NLRP1, and NLRP3 through its SPRY domain to recruit major regulators of autophagy, namely ULK1, BECN1/Beclin 1, and ATG16L1. This process, termed precision autophagy, involves direct recognition of the degradative targets by the core autophagic machinery (Kimura et al., 2015). Although the autophagic components have a general inhibitory effect on the inflammasome, they induce the unconventional secretion of pro-inflammatory cytokine IL1β into the extracellular environment. Therefore, autophagy (in its involvement with inflammasomes and their substrates) appears to play a balancing role in supporting productive inflammatory responses while preventing excessive inflammatory responses and tissue damage. However, the relationship between autophagy and apoptosis is difficult to define. Autophagy may coincide with apoptosis and promote cell death (Mariño et al., 2014; Ji et al., 2021) or antagonize apoptosis to promote cell survival (Bohensky et al., 2007; McKay and White, 2021).

A close correlation between inflammation and autophagy has also been reported, indicating that enhancing autophagy may alleviate inflammation. Autophagy dysfunction in neurons or glial cells is associated with neurodegenerative diseases (Bohensky et al., 2007). In a study of lipopolysaccharide (LPS)-induced neuroinflammation in N9 microglial cells, LPS inhibited the expression of autophagy flux and Vps34 through the activation of PI3KI/AKT/mTOR pathway, whereas rapamycin injection enhanced microglia autophagy and downregulated LPS-induced neuroinflammation (Liu et al., 2019). The authors suggested that promoting the early stages of autophagy could be a potential therapeutic approach for neuroinflammatory diseases. Atherosclerosis is a complex chronic disease caused by the formation of atherosclerotic plaques. The disease is characterized by the upregulation of endothelial dysfunction, leading to an inflammatory response (Li et al., 2015; Zhang et al., 2017). In a study on atherosclerosis, lncRNA-FA2H-2 alleviated the inflammatory responses induced by oxidized-low-density lipoprotein through autophagy flux induction (Guo et al., 2019). In addition, activation of autophagy in mesenchymal stem cells (MSCs) decreased the concentration of inflammatory cytokines when co-cultured MSCs regulated the recruitment and polarization of CD4^+^ T cells *in vitro*. In an *in vivo* study of whether microvesicles of MSCs can act on acute lung injury, investigators demonstrated that MSC microvesicles enhanced autophagy and partially alleviated acute lung injury by delivering miR-100 (Chen et al., 2020a). Therefore, autophagy is hypothesized to be a key regulator of MSC-mediated immune regulation, which may be a potential new strategy to improve the efficacy of MSC-mediated therapy (Cen et al., 2019). In an animal inflammatory model of non-alcoholic steatohepatitis (NASH), ezetimibe ameliorated hepatic steatosis, inflammation, and fibrosis by inducing AMPK-mediated autophagy activation. Human non-alcoholic fatty liver or NASH liver shows decreased autophagic vesicle formation, impaired autophagy pathway, and decreased nuclear TFEB expression. Ezetimibe-induced autophagy markedly blocked NLRP3-inflammasome activation and subsequent IL1β release in macrophages (Kim et al., 2017). Overall, autophagy is closely related to the occurrence of inflammatory reactions, and autophagy dysfunction may induce inflammation. Enhancing autophagy can alleviate inflammation and reduce the level of inflammatory cytokines. While autophagy and inflammation are interdependent processes, macroautophagy is associated with most of the reported suppressive effects on inflammation, and the implications of other types of autophagy in inflammatory reactions are unclear.

## Cannabinoid ameliorates inflammatory diseases by regulating apoptosis

Cannabis contains various complex cannabinoids, such as tetrahydrocannabinol (THC) and CBD, which affect inflammatory and lymphocyte activation pathways in the lymphoid tissue and brain. Therefore, it is increasingly being used in clinical settings. The use of cannabinoids in treating several diseases has been extensively studied in Western countries. Several studies on viral infections have revealed that cannabis inhibits pro-inflammatory pathways in HIV-infected adults and non-human primates infected with SIV (Rizzo et al., 2018; Costiniuk and Jenabian, 2019; Watson et al., 2020). Apoptosis caused the depletion of CD4^+^ and CD8^+^ T cells in HIV-1 infection, leading to an increased risk of infection and increased mortality. In a cohort study of patients with HIV, increased CD4^+^ and CD8^+^ T cell counts were associated with the use of cannabinoids (Keen et al., 2019). In an animal model of SIV-infected rhesus monkeys, the apoptosis of intestinal lymphocytes was reduced after THC treatment, suggesting that chronic THC administration can regulate duodenal T-cell populations, promote Th1/Th2 cytokine balance, and reduce intestinal cell apoptosis (Molina et al., 2014). Overall, cannabinoids exert their classical anti-inflammatory effects on the cells of monocyte lineage in HIV infection while protecting T lymphocytes from apoptosis.

Ulcerative colitis (UC) is a chronic immune-mediated inflammatory bowel disease that involves the dysfunctional immune response to the normal microbiota and dietary contents in the gastrointestinal tract. The inflammatory cascade activates T cells, which secrete excessive amounts of pro-inflammatory cytokines, including IL-1, IL-6, and TNF-α, thereby damaging healthy tissues. Endoplasmic reticulum stress and unfolded protein reaction are closely related to cell apoptosis, autophagy, and inflammatory reaction in the pathogenesis of UC (Hetz, 2012; Cao, 2016). Severe or persistent endoplasmic reticulum stress eventually triggers the internal apoptosis pathway, leading to cell death and aggravating UC (Zhang et al., 2015). In an *in vitro* study on inflammatory bowel disease, CBD reduced the production of reactive oxygen species and lipid peroxidation in cells, thereby reducing the occurrence of apoptosis (Borrelli et al., 2009; Esposito et al., 2013). Meanwhile, some authors suggest that CBD may indirectly activate the CB1 receptor in the intestine, thereby reducing intestinal motility and relieving UC symptoms [ (Capasso et al., 2008). Taken together, although there is definite evidence that CBD has a therapeutic effect in autoimmune diseases, its action mechanisms need to be explored further.

Several authors have confirmed that A2A, A2B, and A3 receptors participate in the antiapoptotic effect of ATP through MEK/ERK1/2, PKA, and NOS pathways. The selective antagonists of A2A, A2B, and A3 receptors limit the antiapoptotic effect of ATP against hypoxia (Feliu et al., 2019). In the LPS-induced acute lung injury animal model, CBD reduced the migration of leukocytes to the lung, thereby decreasing the concentration of albumin and pro-inflammatory cytokines in bronchoalveolar lavage fluid. The use of an A2A antagonist restored the inflammatory response, suggesting that CBD may play a role by binding with the A2A receptor (Ribeiro et al., 2012). In addition, in the inflammatory model of demyelinating disease induced by encephalomyelitis virus, the application of CBD reduced leukocyte infiltration and activated microglia in the cerebral cortex, suggesting that CBD can limit inflammatory response by increasing adenosine signal transduction (Longoria et al., 2022). Overall, we suggest that CBD can inhibit inflammation; however, whether it plays an anti-inflammatory role by binding to adenosine receptors to inhibit apoptosis needs further investigation.

Liver fibrosis is the basic pathologic change in the liver caused by chronic liver diseases. The activation of hepatic stellate cells (HSCs) leads to the accumulation of scar matrix and fibrotic liver. A basic study on HSCs revealed that CBD induced downstream activation of the IRE1/ASK1/c-Jun N-terminal kinase pathway that promoted apoptosis, leading to HSC death. The authors suggested that CBD can be used as a potential therapeutic agent for chronic hepatitis that leads to liver fibrosis by selectively inducing apoptosis of activated HSCs (Lim et al., 2011).

## Cannabinoids promote autophagy and ameliorate chronic inflammatory diseases

Autophagy, a lysosomal catabolic process, is critical to cell homeostasis and is crucial to the neuroprotection of the central nervous system. Autophagy defects are observed in many neurodegenerative diseases (Baron et al., 2019). However, abnormal autophagy activation may aggravate the extensive ischemic and inflammatory processes caused by nerve traumatic diseases (Lu et al., 2019). CBD, a non-psychoactive cannabinoid, is becoming a promising therapeutic agent for mental disorders and inflammatory diseases (Campos et al., 2016). CBD has antidepressant (Linge et al., 2016), antiemetic (Rock and Parker, 2015), neuroprotective (Schiavon et al., 2014; Silveira et al., 2014), anticonvulsant (Pelz et al., 2017), antianxiety (Crippa et al., 2011), and antipsychotic effects (Rohleder et al., 2016). CBD has pharmacological properties in the treatment of neurological diseases. Therefore, the mechanism of CBD-mediated potential autophagy activation needs to be explored further. The neuroprotective effects of autophagy occur because it reduces the effects of inflammation (Lowin et al., 2020).

Neuronal cell death caused by chronic inflammation, which is induced by dysfunctional mitochondria, is considered the main cause of neurodegenerative diseases (Johnson et al., 2021). When mitochondrial dysfunction occurs in cells, accurate autophagy can prevent the occurrence of chronic inflammation and delay degenerative diseases. PPAR agonists restore autophagy, enhance mitochondrial *ß*-oxidation, and stimulate mitochondrial biosynthesis; therefore, they can be potentially used to treat patients with steatohepatitis (caused by glycogen storage disease type Ia) (Zhou et al., 2019). CBD mediates the functions regulated by PPARα and *?* receptors and plays neuroprotective, anti-inflammatory, and metabolic roles (O'Sullivan, 2016). PPARγ agonists have been shown to reduce inflammatory processes associated with chronic and acute nerve injury (Kapadia et al., 2008). CBD can bind and activate PPARγ *in vitro* (Hegde et al., 2015); it can also protect rats from neurological and inflammatory damage caused by *ß*-amyloidosis (Esposito et al., 2011). In addition to its role in inhibiting inflammation, some studies have evaluated whether CBD treatment is feasible for chronic inflammatory pain (Britch et al., 2020). In another study on neurodegenerative diseases, CBD played an important role in autophagy activation by regulating ERK1/2 and AKT kinase phosphorylation and participating in ULK1 (Vrechi et al., 2021). In this study, low doses of CBD promoted autophagy flux without affecting cell viability, and CB1, CB2, and TRPV1 receptors mediated CBD-induced autophagy, which is crucial to maintain neuronal activity and function.

CBD plays a role in the treatment of neurodegenerative diseases by promoting autophagy, participating in homeostasis, and regulating the circulation and degradation of cellular proteins through the lysosome degradation pathway; however, it exerts the opposite effect in autoimmune diseases. RA is characterized by an anoxic environment in the joints accompanied by mitochondrial dysfunction. The compounds that inhibit autophagy (chloroquine or hydroxychloroquine) are being clinically used to treat RA. In an animal model of RA chronic inflammation, CBD showed anti-inflammatory and analgesic effects. In some studies, immune cells and synovial fibroblasts were reduced following CBD treatment to alleviate inflammation (Hammell et al., 2016; Lowin et al., 2020). In addition, CBD may also interact with antirheumatic drugs, such as methotrexate or JAK inhibitors to increase the absorption of cytotoxic chemotherapy drugs (Fraguas-Sánchez et al., 2020). CBD is feasible as an adjuvant treatment for RA and can be used in combination with other antirheumatic drugs. The underlying mechanism may involve the binding of CBD to β2 adrenergic receptors and then activating downstream *ß*-arrestin2 to inhibit autophagy (Lowin et al., 2019; Chen et al., 2020b)].

### Effect of apoptosis on the progression of cancer

The occurrence and development of cancer represent the imbalance between cell proliferation and cell death, indicating that the rate of proliferation and mutation of tumor cells exceeds that of dead cells. The early understanding of cancer mainly revolved around cell oncogenes, cell proliferation, and cell transformation. Apoptosis can be induced as a part of cancer treatment because of the detection of DNA fragmentation and changes after chemotherapy (Wyllie, 1980). Cell apoptosis is a common tumor inhibition mechanism. The molecular mechanism of apoptosis has been elucidated in detail. The key is to activate caspases, which can activate the internal pathway through mitochondrial outer membrane permeabilization or activate the death receptors, such as Fas, DR4, and DR5, on the cell surface by their death-inducing ligands (FasL and TRAIL). In recent decades, research on cancer treatment has mainly focused on the development of drugs and radiotherapy to increase tumor cell death, reduce tumor volume, and block invasion. Although many survival-promoting pathways have become established drug targets in oncology, it is significant to recognize that drugs ultimately induce apoptosis through the core apoptosis pathway (Hassan et al., 2014; Baig et al., 2016). The discovery of the BCL-2 gene in patients with follicular lymphoma can be instrumental in controlling cancer growth by promoting cell apoptosis (Pekarsky et al., 2018). Moreover, FDA has approved a BCL-2 targeted drug that promotes cell apoptosis and controls the growth of cancerous cells. Selective Bcl-2 inhibitors were used in a clinical study of leukemia or non-solid tumors (Némati et al., 2014; Leverson et al., 2017; Casara et al., 2018). Bcl-xL inhibitors were used in combination with traditional chemotherapeutic drugs for treating some solid tumors (US National Library of Medicine, 2019). The inhibitor of apoptosis (IAP) proteins is overexpressed in several malignancies and negatively affect prognosis by preventing caspase activation, thereby promoting tumor cell survival (Fulda and Vucic, 2012). Bcl-2-targeting and IAP-targeting inhibitors target the intrinsic pathways of apoptosis. The exogenous pathways are activated by extracellular signals that activate apoptosis-promoting death receptor (DR)- and TNF-receptor superfamily (TNFR) transmembrane proteins (Green and Llambi, 2015). Death receptors include TNFR1, Fas (CD95 and APO-1), DR3, DR4 (TRAILR1), DR5 (TRAILR1), and DR6. Mapatumumab, a DR4-receptor agonist, in combination with chemotherapy has shown limited clinical benefit in phase I clinical trials in patients with non-small cell lung cancer, colorectal cancer, and other solid tumors (Hotte et al., 2008; Trarbach et al., 2010; von Pawel et al., 2014). Lexatumumab, a DR5 agonist, in combination with chemotherapy stabilized the growth of advanced solid tumors but some adverse reactions were also recorded (Merchant et al., 2012). Therefore, targeting the apoptotic pathway in tumor cells is an effective anticancer strategy. Further, promoting the death of tumor cells helps to improve the clinical status of patients and reduce the chance of tumor recurrence.

In addition to cancer cells, tumor lesions also contain other cell types, such as endothelial cells and cancer-related fibroblasts, which inhibit the development of cancer (Hanahan and Weinberg, 2011). In addition, innate and adaptive immune cells, which grow and infiltrate tumor tissues to fight tumors, combine with these cells to form the tumor microenvironment (TME). Although voluntary apoptosis constitutes a common tumor suppressor mechanism, apoptosis may promote tumor cell survival and resistance to therapy by modulating the TME. The complex interrelationship between cellular and non-cellular components of TME coordinates tumor formation, progression, invasion, and response to therapy (Elia and Haigis, 2021), which may determine its different effects in the face of apoptotic regulation. This phenomenon may be related to the subsequent phagocytosis of apoptotic cells. Apoptotic cells can be phagocytosed by cells in neighboring tissues, especially macrophages. Macrophages can recognize the “find me” (such as phosphatidylcholine, 1-phosphosphingosine, and chemokine CX3CL-1) and “eat me” (phosphatidylserine) signals on the surface of apoptotic cells (Boada-Romero et al., 2020). Tumor-associated macrophages (TAMs) occupy the majority of TME in several malignant tumors; TAM accumulation is closely related to tumor growth and angiogenesis. Apoptosis-driven tissue repair and regeneration responses may be involved in generating and supporting TME (Ford et al., 2015; Ichim et al., 2015; Gregory et al., 2016). Apoptotic cells in the TME generate an oncoregenerative niche, which refers to cellular and tissue programs that are activated when these cells promote tumorigenesis and malignant disease progression through apoptosis-driven regeneration and tissue repair mechanisms, thereby promoting tumor expansion and invasion while inhibiting antitumor immunity (Gregory and Paterson, 2018). The main function of TAMs in cancer is to support tumor growth through various mechanisms, including activation of angiogenesis, production of growth and survival factors, support of invasion and metastasis, and inhibition of antitumor immunity (Balkwill and Mantovani, 2012; Weigert et al., 2016). Apoptotic cells induce M2 -like activation of macrophages. Moreover, they are easily engulfed by M1 macrophages and can inhibit their antitumor activity (Voss et al., 2017). However, more studies are needed to understand how apoptosis drives the M2-like phenotype of TAMs in the oncoregenerative niche. Lactic acid produced by TAMs may be necessary to promote tumor activation by polarizing to an M2-like phenotype.

Overall, apoptosis may be a “double-edged sword” for tumor tissue. On the one hand, it inhibits tumors by removing malignant or precancerous cells, and on the other hand, it promotes tumor progression by stimulating the repair and regeneration response in the TME.

### Role of autophagy in the regulation of cancer

Autophagy maintains a steady state in the single cell, and damaged organelles, cytoplasmic substances, and misfolded proteins are subjected to lysosome degradation. Apoptosis occurs in multicellular organisms. The occurrence of autophagy-induced cell death represents the failure of the cell to resist a lethal factor. This form of cell death is often referred to as cell death associated with autophagy-independent caspases (Shen et al., 2012). Autophagy pathway relies on the formation of autophagosomes, which depends on the recruitment of various autophagy-associated (ATG) proteins. This activation process begins with the formation of a pre-initiation complex composed of kinases UNC-51-like kinase 1 (ULK1), FIP200, and ATG13, which activates downstream initiation complexes composed of Beclin 1, Class III PI3K (Vps34), and protein kinase Vps15. This initiation complex leads to the production of the lipid phosphatidylinositol 3-phosphate (PI3P), which activates ATG5-12 and LC3-PE conjugation systems (He and Levine, 2010). Finally, LC3 is conjugated with phosphatidyl ethanolamine (PE) to form the LC3-PE conjugate (also known as LC3-II) to aid the formation of autophagosomes. Pre-initiation complex formation is regulated by two major metabolic checkpoints, mammalian target protein complex 1 (mTORC1) and AMP-activated protein kinase (AMPK) (Hardie, 2012; Laplante and Sabatini, 2012). Initiation complex activity is negatively regulated by several independent signaling pathways (including the growth factor and PI3K-AKT signaling pathways) (Wang et al., 2012), antiapoptotic proteins Bcl-2 and Bcl-xL (Pattingre et al., 2005), death-related protein kinases, and JNK family kinases (Wei et al., 2008).

Autophagy is a multistep lysosomal degradation pathway that supports nutrient cycling and metabolic adaptation. In the early stages of cancer, autophagy may play a role in limiting tumorigenesis. However, autophagy can help tumor tissue respond to intracellular and environmental stresses, such as hypoxia, nutrient limitations, and anticancer treatments during cancer progression and metastasis, thereby facilitating tumor progression (Degenhardt et al., 2006). In the early stages of tumorigenesis, autophagy acts as a control mechanism to protect tissues from damage, prevent tumorigenesis and genetic accumulation and inhibit cell apoptosis by removing damaged organelles and defective proteins (Chavez-Dominguez et al., 2020). When the pressure of the living environment of tumor cells is too severe, autophagy can repair damaged DNA and organelles, thus maintaining tumor expansion and survival (Eskelinen, 2011).

Therefore, autophagy may also play a dual role in cancer by either enhancing tumor tolerance or regulating self-sacrifice to preserve tumor tissue integrity (Towers et al., 2020; Verma et al., 2021). The role of autophagy in cancer treatment depends on specific stimuli, tumor cell types, and the severity of the injury and is not completely determined by the progression of the tumor (Qu et al., 2020). Although autophagy plays a paradoxical role in the treatment of tumors, understanding the pathophysiology and functional correlation of autophagy in tumors is still crucial to avoid drug resistance and enhance the anticancer therapeutic efficacy. For example, although there are many examples suggesting that autophagy has a cell-protective function in lung cancer (Cheng et al., 2013), the cytotoxic function of autophagy has also been reported (Shin et al., 2012). Pemetrexed is a folate antimetabolite approved for the treatment of non-small cell lung cancer and has been shown to regulate autophagy. This drug in combination with a multikinase inhibitor sorafenib, increased the levels of AKT, p70 S6K, and/or phosphorylated mTOR, thereby enhancing tumor killing by promoting toxic forms of autophagy (Bareford et al., 2011). Cisplatin is also a commonly used chemotherapeutic drug in the treatment of lung cancer, but the long-term treatment causes tumor resistance; the potential mechanism involved a reduction in autophagy (Sirichanchuen et al., 2012). Mechanically, long-term cisplatin treatment causes overexpression of Bcl-2, and the results of *in vitro* cell line studies have suggested that cisplatin resistance can be reversed by using triflurazine to induce autophagy. In some studies on radiation therapy for cancer, the autophagy inhibitor, 3-methyladenine, induced radiation resistance in liver cancer cells (Kuwahara et al., 2011)]. Rapamycin, as an autophagy inducer, enhanced the sensitivity of glioma-initiating cells to radiation (Zhuang et al., 2011).

**FIGURE 3:**
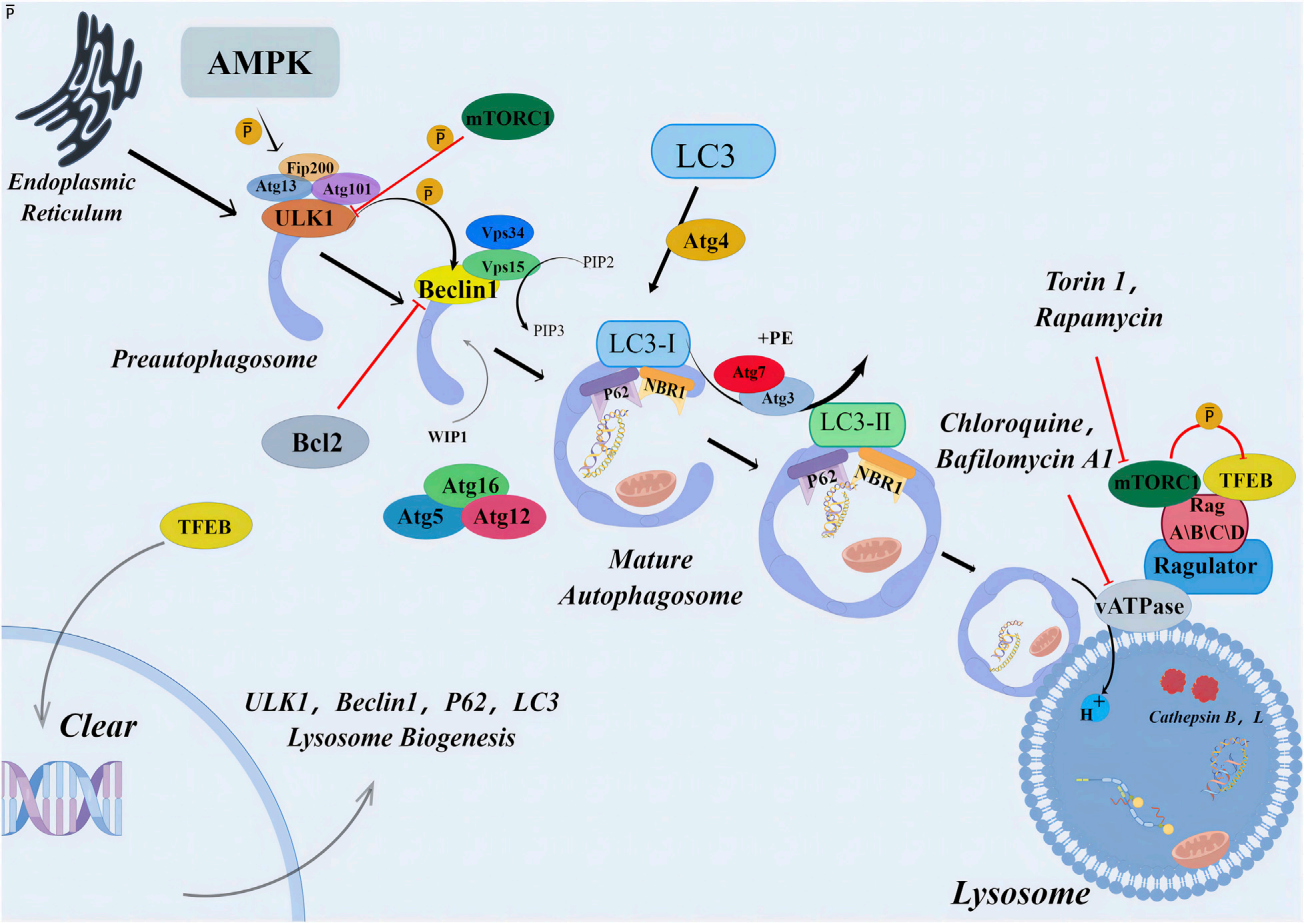
Primary mechanism of autophagy. The autophagic signaling pathway. Under metabolic stress, AMPK activation and/or mTORC1 inhibition lead to the activation of the preinitiation complex (ULK1, FIP200, and ATG13). The latter activates the initiation complex (beclin 1, VPS15, and VPS34) that generates PI3P and recruits ATG7 to the phagophore. ATG7 initiates two conjugation pathways necessary for membrane elongation and closure of the autophagosome. In the ATG5-ATG12 conjugation pathway, ATG12 is sequentially transferred to ATG7, ATG10, and ATG12. The ATG5-12 conjugate recruits ATG16L and forms a complex necessary to stabilize the phagophore and to complete the second conjugation pathway. In the LC3-PE pathway, LC3 is cleaved by ATG4 and sequentially conjugated to ATG7 and ATG3. The ATG5-ATG12-ATG16 L complex carries out the final step by transferring LC3 to PE to form an LC3-PE conjugate (also called LC3-II). LC3-II binds to the autophagosomal membrane, and form autolysosome. AMPK, AMP-activated protein kinase; Atg, autophagy related gene; Bcl2, antiapoptotic protein; Beclin1, myosin-like BCL2 interacting protein; LC3 (MAP1LC3), microtubule-associated proteins light chain three; mTORC1, mammalian target of rapamycin complex one; p62, p62 protein; PIP, polyphosphoinositides; Rag, Rag proteins (RagA–D); TFEB, transcription factor EB; ULK, Unc-51-like kinase; vATPases, vacuolar-type adenosine triphosphatases.

In addition to the interaction of Beclin-1 with class III phosphatidylinositol-3-kinase Vps34 to initiate the classical pathway of autophagy, non-classical pathways also exist. This implies that autophagy can be regulated from multiple directions. Some authors used resveratrol to inhibit the proliferation of human breast cancer MCF-7 cell line and promote cell death and found that typical autophagy biomarkers such as LC3-I and LC3-II accumulated in the presence of lysosome inhibitor E64 and gastric enzymostatin A (Scarlatti et al., 2008). Moreover, knockout of neither Beclin-1 nor hVPS34 gene eliminated resveratrol-induced autophagosome formation, suggesting the existence of a non-classical pathway independent of tumor suppressor Beclin-1 (Figure 3).

Autophagy and apoptosis lead to cell death in a parallel manner. However, in some cases, autophagy can also be used as a part of the apoptosis program and a backup cell death mechanism after the inhibition of caspases (Denton et al., 2012). When stress-induced apoptosis is blocked, autophagy can be another mechanism of cell death. Both these processes share key metabolic regulators, indicating that these pathways are used under similar cell death or survival pressures.

The role of apoptosis in cancer tissues and TME is contradictory due to their complexity. Several new forms of autophagy, such as secretory autophagy, regulate cancer progression, which realizes intercellular communication through the release of tumor-promoting substances in TME [ (New and Thomas, 2019). Simultaneously, complex crosstalk happens between autophagy and epithelial-to-mesenchymal transformation (EMT), which enables cancer cells to develop invasive phenotypes and metastatic potential (Singla and Bhattacharyya, 2017). Several components of autophagy pathways also mediate many non-autophagic functions. This shows that autophagy plays a role in many ways, including building complex signal networks with other cell elements, integrating various signals in the TME, and regulating the fate of cancerous and other cells in the microenvironment. Therefore, understanding the mechanism of autophagy inhibition in complex systems is crucial for developing strategies to use autophagy pathways as targets for controlling tumor growth.

## Potential application of the endogenous cannabinoid system (ECS) in cancer

In the past decade, ECS has become the focus of anticancer therapies. ECS refers to the complex network of cannabinoid receptors, endogenous cannabinoid ligands, enzymes that drive its biosynthesis, degradation, and transportation, and all cells and neural pathways involved in endogenous cannabinoid signal transduction. Presently, ECS is thought to be related to the important processes of controlling the body, assisting and maintaining the homeostasis of the body, which explains its uncertain role in tumorigenesis and tumor inhibition.

CB1 and CB2 are the main receptors in the ECS. They belong to the extensive class A rhodopsin-like G protein-coupled receptor family, and their main functions include completing the transduction of signal molecules from extracellular space to various cells (Pertwee et al., 2010). The signal transduction of endocannabinoids is different from that of neural signal transduction. The role of endogenous cannabinoids is mainly limited to their biosynthesis and storage in synaptic vesicles in advance and released to the receptors when required (Lu and Mackie, 2016). Endogenous and exogenous cannabinoids have other in the central nervous system and tumor tissues (Soderstrom et al., 2017), including transient receptor potential channels, ligands, voltage-gated ion channels, and other orphan G protein-coupled receptors such as GPR55, GPR18, and GPR119. The exogenous cannabinoids may cause the activation, antagonism, or reverse activation of cannabinoid and non-cannabinoid receptors because of the differences in the abundance of receptors and levels and activities of endogenous ligands (Pertwee, 2015). This may also be one of the reasons for the inconsistent effects of exogenous cannabinoids observed in many empirical studies.

CB1 and CB2 and other members of the endogenous cannabinoid-like system have become novel targets to treat various cancer subtypes because of their dual roles in tumorigenesis and inhibition of tumor growth and metastasis. Although the clinical use of cannabinoids has been widely recorded in palliative treatment, clinical trials on their use as an anticancer drug are still in progress. Cannabinoid receptors and endocannabinoids are usually upregulated in tumors, and their expression levels may be related to tumor invasiveness (Malfitano et al., 2011). Some authors have revealed that the loss of CB1 accelerates tumor growth (Wang et al., 2008), and higher levels of endogenous cannabinoid in the body lead to the reduction of precancerous lesions (Izzo et al., 2008), which confirms the theory of CEDS (occurrence of some diseases is related to the disorder of ECS signal transduction). The relationship between the distribution and expression of CB1 and CB2 on tumor cells and the tumor itself is not clear. Some authors believe that the loss of CB1 and/or CB2 expression accelerates tumor growth; however, it is likely to be related to the type of tumor cells (Pagano and Borrelli, 2017). More clinical trials are needed to confirm the accuracy of results to better understand the relationship between ECS and neoplasmic diseases.

### Cannabinoid compounds improve cancer treatment by regulating apoptosis and autophagy

The endocannabinoids can bind to very rich channel sites in the body, which coincide with the receptors that simultaneously mediate the pathological process of cancer, such as PPAR. Targeted activation of PPARγ can inhibit cell proliferation and induce programmed cell death (apoptosis and autophagy) (Campbell et al., 2008). In an *in vitro* study, CBD could activate the apoptosis of lung cancer cells after binding to the PPARγ receptor (Ramer et al., 2013). In another study on human cervical cancer cells (treated with met-AEA in advance), the occurrence of apoptosis depended on the production of PGD 2 and PGJ 2 and the activation of the PPARγ receptor (Eichele et al., 2009). In addition to exogenous intake, regulating enzymes are also an important link in regulating endogenous cannabinoid levels. Fatty acid amidohydrolase (FAAH) and monoacylglycerol lipase are the two most important endocannabinoid hydrolases. In non-melanoma skin cancer cells, the use of FAAH inhibitor URB597 increased the apoptosis-promoting effect of AEA (Kuc et al., 2012). Moreover, URB597 can increase the autophagic toxicity of AEA on neuroblastoma cells by inhibiting the hydrolysis of AEA by FAAH (Hamtiaux et al., 2011). These studies suggest that URB597 can increase cellular endocannabinoid levels and enhance the interaction between endocannabinoid and its molecular target by reducing the activity of hydrolases. In addition, several studies have suggested that endocannabinoids can play an anticancer role through the endoplasmic reticulum stress pathway. In hepatocellular carcinoma cells (HEPG1), the use of plant cannabinoids increased the phosphorylation of eIF2α and expression of CHOP10 transcript TRB3 (Vara et al., 2013). TRB3 is necessary to inhibit Akt/mTORC1 signal transduction and induce autophagy and apoptosis (Salazar et al., 2009), indicating that exogenous cannabinoid contributes to tumor autophagy and apoptosis. Synthetic cannabinoids have also been used in several studies. In human glioblastoma cell lines, the ability of THC to inhibit cell growth and induce apoptosis was associated with the activation of CB1 and CB2 receptors, which was validated by the use of selective receptor antagonists or by the silencing of receptor expression using specific small interfering RNA (Kolbe et al., 2021). Neuroblastoma is one of the most common childhood cancers, CBD induces apoptosis of neuroblastoma cells by activating serotonin and vanillin receptors. In this process, miRNA hsa-mir-1972 is upregulated, resulting in decreased expression of apoptosis-related genes BCL2L1 and SIRT2 and regulating the apoptosis of neuroblastoma cells (Alharris et al., 2019).

Synthetic cannabinoid receptor agonists are more widely used in research than naturally occurring cannabinoids. Synthetic cannabinoid receptor antagonists AM251, SR144528, and AM630 inhibit osteoclast formation and bone resorption *in vitro*. Cannabinoid receptor blockade caused osteoclast inhibition mostly through apoptosis of osteoclasts because AM251 and SR144528 markedly enhanced apoptosis in mature rabbit osteoclasts and RANKL-induced (Idris et al., 2005)]. The effect of the CB receptors on cell apoptosis is gradually being determined. In a study on liver cancer, researchers found that Δ9-THC and JWH-015 (a CB2 cannabinoid receptor-selective agonist) reduced the viability of the human hepatocellular carcinoma cell lines HepG2 and HuH-7, an effect that relied on the stimulation of CB2 receptor (Vara et al., 2011). JWH-015 also reduced pain in patients with bone cancer by improving impaired autophagy flux (Mao et al., 2019). Arvanil (N-arachidonoylvanillamine), a compound similar to AEA (Di Marzo et al., 2002), induced apoptosis of Jurkat cells, which was essentially mediated through mechanisms typical of type II apoptotic cells and involved activation of the death-inducing signaling complex and caspase-8. This arvanil-induced apoptotic activity is independent of CB1 and has important implications in the development of antitumor drugs (Sancho et al., 2003). Some authors have reported that a synthetic cannabinoid WIN55212-2 can induce autophagy and apoptosis in human colorectal cancer and mantle cell lymphoma cells; this activity is related to the induction of cytotoxic endoplasmic reticulum stress (Wasik et al., 2011; Pellerito et al., 2014).

**FIGURE 4:**
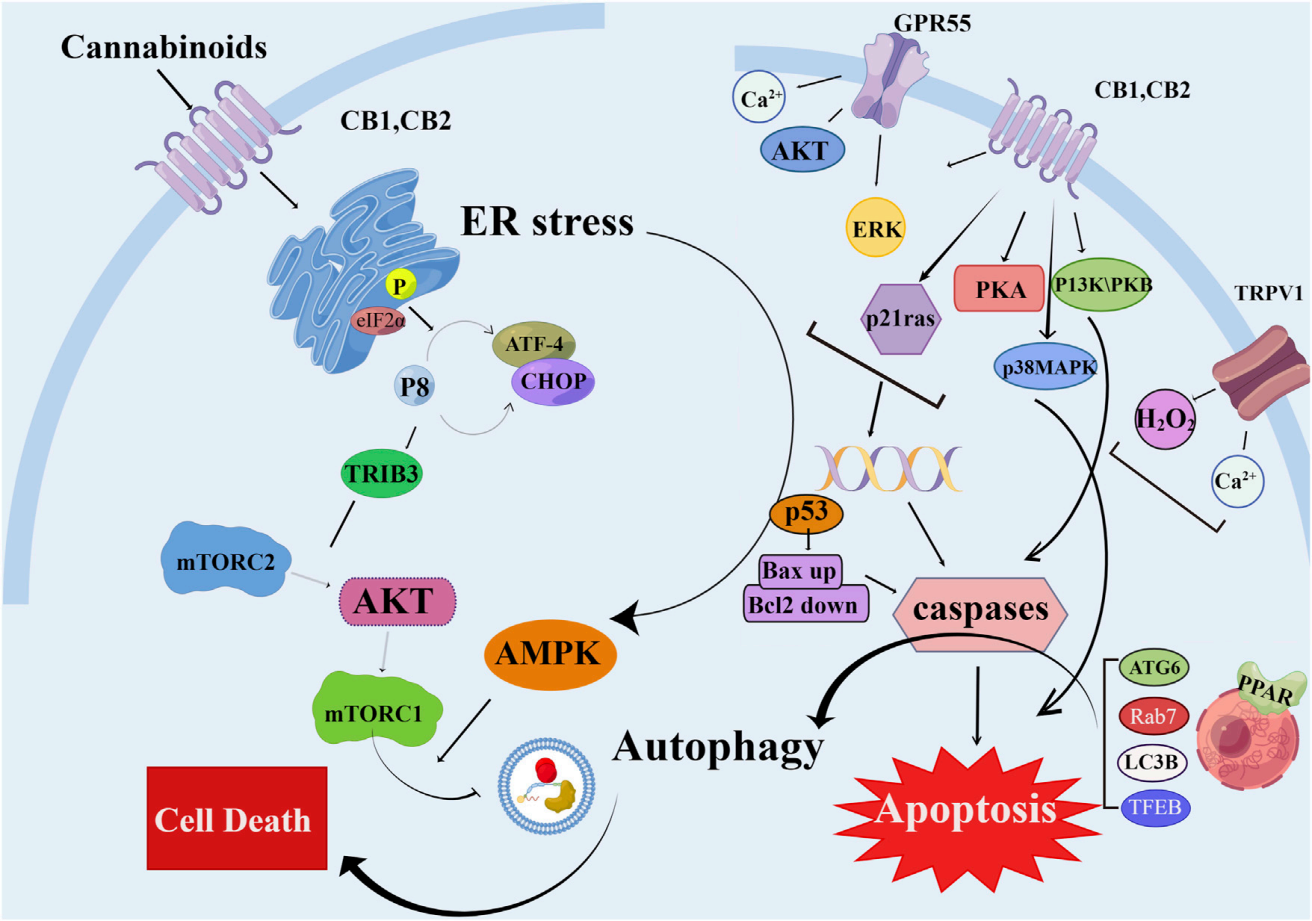
Schematic diagram of different mechanisms/signaling pathways of cannabinoids affecting autophagy and apoptosis. The mechanism of cannabinoid-induced apoptosis and autophagy in cell is depicted. AKT, protein kinase B; AMPK, AMP-activated protein kinase; ATF-4, activating transcription factor 4; Bax, pro-apoptotic protein; Bcl2, antiapoptotic protein; CB1/2, cannabinoid receptor 1/2; CHOP, C/EBP homologous protein; elF2α, eukaryotic translation initiation factor 2α; ERK, extracellular regulated kinase; GPR55, G protein-coupled receptor 55; H_2_O_2_, hydrogen peroxide; mTORC2, mammalian target of rapamycin complex two; p53, p53 protein; p21 ras, p21 ras protein; p38MAPK, p38 mitogen activated protein kinase; PI3K, phosphatidyl inositol three kinase; PKA, protein kinase A; TFEB, transcription factor EB; TRIB3, tribbles-homologue three; TRPV1, transient receptor potential cation channel V1.

Cannabinoids bind to CB1 and CB2 receptors to inhibit energy metabolism in pancreatic cancer cells and induce AMPK-dependent autophagy (Dando et al., 2013). A new synthetic cannabinoid (CB83) has been used on human HT-29 colorectal adenocarcinoma cells; it induced the activation of endoplasmic reticulum stress-related signaling pathways by increasing ceramide production, ultimately leading to the activation of the intrinsic apoptotic pathway and inhibiting the proliferation of cancer cells (Cerretani et al., 2020) (Figure 4).

Taken together, cannabinoids have great prospects in cancer treatment. We have reviewed the available information on natural or synthetic cannabinoid compounds that target cancer cell apoptosis and autophagy. However, it can bind to receptors other than CBR1 and CBR, or directly act on some pathways to exert its antitumor effects. CBD, a plant-derived cannabinoid, can combine with a variety of receptors not involved in the pathology of mental disorders; therefore, it has attracted much attention as an anticancer drug.

## Conclusion and outlook

PCD has been increasingly implicated as the gatekeeper of cell fate, with decisive roles in various diseases including inflammatory diseases, autoimmune diseases, degenerative diseases, and cancer. The role of cell death in the pathophysiology of inflammation and cancer needs to be explored further. Several *in vitro*/*in vivo* studies on exogenous cannabinoids have indicated that cannabinoids can affect the key functions of cells in inflammatory or cancer diseases, such as proliferation, migration, cytokine formation, differentiation, autophagy, and apoptosis. The diversity of cellular receptors of cannabinoid ligands may explain the wide range of biological effects of cannabinoid drugs in several diseases. More information on cannabinoid CB1/CB2-independent targets is needed to determine their *in vitro* effects and tolerance/resistance mechanisms and to properly interpret the results of available studies. Previous studies have shown that cannabinoids have an immunosuppressive effect on the immune system. Cannabinoid ligands inhibit phagocytosis, cell proliferation, antigen presentation, and other properties of immune cells, and cannabinoid may be a safe and effective antitumor drug. Nevertheless, clinical studies to evaluate the effect of cannabinoids on the human body are limited. Only animal models or *in vitro* cell lines have been used to evaluate the feasibility of cannabinoid treatment and large-scale clinical trials have not been conducted. Therefore, the results may vary in the clinical setup.

The prospects of cannabinoid treatment in inflammatory and cancer diseases are worth exploring. The influence of cannabinoids on cell fate should be explored further. A detailed understanding of the regulation of autophagy and apoptosis by cannabinoids will not only improve the understanding of the biology of the disease but will also be crucial in finding better therapeutic targets, thereby generating new combined therapies.
